# Modulation of CCR2/CCL2 molecular axis in the expansion and rupture of abdominal aortic aneurysms

**DOI:** 10.3389/fcvm.2026.1772497

**Published:** 2026-02-17

**Authors:** Ryan Wahidi, Santiago Elizondo-Benedetto, Ryan Catlett, Bera Koklu, Mohamed A. Zayed

**Affiliations:** 1 Division of Vascular Surgery, Department of Surgery, Washington University School of Medicine, St. Louis, MO, United States; 2 Cardiovascular Research Innovation in Surgery and Engineering Center, Department of Surgery, Washington University in St. Louis, St. Louis, MO, United States; 3 Department of Radiology, Washington University School of Medicine, St. Louis, MO, United States; 4 Division of Molecular Cell Biology, Washington University School of Medicine, St. Louis, MO, United States; 5 Division of Surgical Sciences, Department of Surgery, Washington University School of Medicine, St. Louis, MO, United States; 6 Department of Biomedical Engineering, McKelvey School of Engineering, Washington University School of Medicine, St. Louis, MO, United States; 7 Department of Surgery, Veterans Affairs St. Louis Health Care System, St. Louis, MO, United States

**Keywords:** aortic aneurysm, aortic rupture, CCR2, inflammation, inhibitor, molecular targeting

## Abstract

The CCR2/CCL2 molecular axis is a critical mediator of abdominal aortic aneurysm (AAA) pathogenesis. It has been demonstrated to drive chronic inflammation, extracellular matrix degradation, and vascular remodeling through the recruitment and activation of monocytes/macrophages and other immune cell types. Pre-clinical studies demonstrate that CCR2 inhibition reduces AAA formation, expansion, and progression in animal models. Emerging imaging techniques have validated CCR2 as a biomarker for AAA instability in humans. Although clinical trials targeting CCR2 are currently limited in number, ongoing translational studies highlight that CCR2 blockade is a promising therapeutic strategy to mitigate AAA expansion and the risk of rupture. This review underscores the potential of CCR2-targeting interventions to fill a critical unmet need to develop effective medical therapies for longitudinal clinical AAA management.

## Introduction

Abdominal aortic aneurysms (AAAs) are progressive localized dilations of the abdominal aorta, typically defined as having a diameter ≥3.0 cm (1, 2). AAAs carry a substantial risk of rupture and rupture events are associated with a high mortality rate of 80%–90%, resulting in approximately 200,000 annual deaths globally (3–5). The majority of AAAs are incidentally detected during imaging performed for unrelated reasons; however, many can be missed and remain undiagnosed until they rupture (6, 7). AAAs predominantly impact older males, with the risk factors including smoking, hypertension, hyperlipidemia, white race, and family history (1, 7). Diagnosis is primarily dependent on imaging, with ultrasound as the gold standard for the initial screening, often followed by computed tomography (CT) or CT angiography, whereas a physical examination has limited sensitivity (8). Management of an AAA is primarily determined by the aneurysm’s size and growth rate. Small aneurysms are monitored with serial imaging and “expectant management,” whereas large aneurysms (≥5.5 cm in males and ≥5.0 cm in females) or those expanding rapidly require elective open or endovascular surgical repair (1–4, 6). Beyond these interventions, there are currently no approved pharmacological therapies to halt aneurysm expansion or prevent rupture events. Furthermore, the lack of reliable diagnostic markers for predicting AAA rupture risk underscores the critical need to better understand the molecular mechanisms driving AAA progression and identify novel theranostic targets (9).

AAA formation is primarily driven by chronic vascular inflammation, leading to elastin degradation, vascular smooth muscle cell (VSMC) loss, fibroblast activation, and phenotype switching (3, 10, 11). Key inflammatory cells, such as neutrophils, monocytes/macrophages, and T cells, progressively accumulate within the aortic wall and secrete various pro-inflammatory cytokines and chemokines, contributing to extracellular matrix (ECM) imbalance through the release of matrix metalloproteinases (MMPs), specifically MMP-2 and MMP-9 (3, 12). These molecular mediators orchestrate the remodeling process that underlies AAA expansion and increased risk of rupture over time (13).

**Figure 1:**
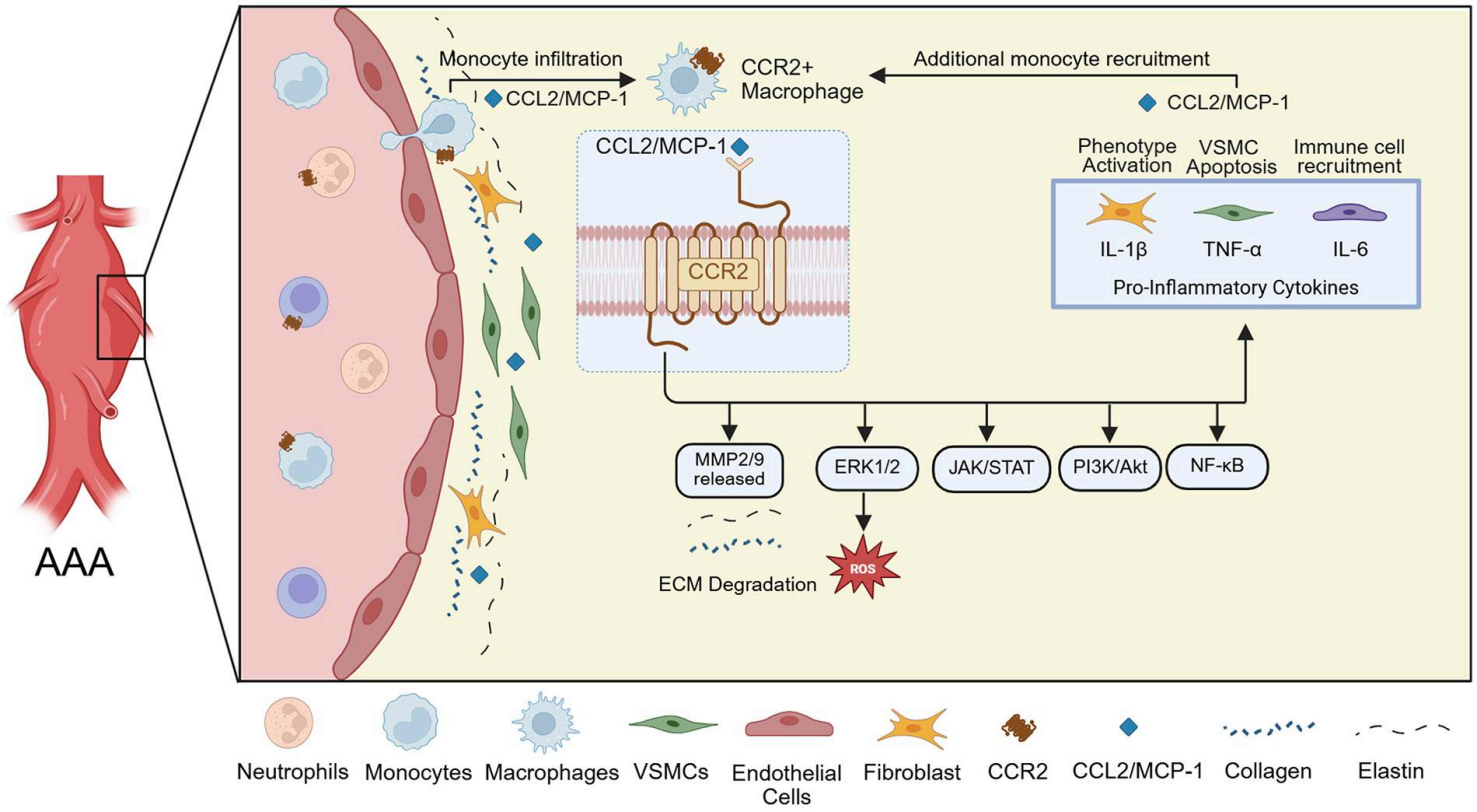
Schematic representation of CCR2+ monocyte recruitment and downstream effects in the AAA wall. The inflammatory environment is associated with elevated MMP-2 and MMP-9 expression in wall tissue, leading to ECM degradation, and the pro-inflammatory cytokines IL-1β, TNF-α, and IL-6 maintain further CCR2+ monocyte recruitment.

Chemokines, in particular, are small signaling proteins that play a vital role in vascular inflammation by mediating leukocyte recruitment to sites of tissue injury (14). These molecules interact with chemokine receptors, typically G protein-coupled receptors, to form chemotactic gradients that guide immune cell migration (14). Chemokines are classified into structural families, most notably CC and CXC, based on the arrangement of disulfide bonds on their conserved cysteine residues. In the context of AAA development, the C-C motif chemokine ligand and receptor type 2 (CCL2 and CCR2, respectively) represent a key molecular axis in the early inflammatory cascade (14–16). CCL2 (also known as monocyte chemoattractant protein-1 or MCP-1) is secreted by activated endothelial cells, fibroblasts, VSMCs, and macrophages within the aortic tissue in response to vascular injury (17). Bone-marrow-derived, circulating immune cells expressing CCR2, especially monocytes, are drawn to sites of inflammation by the CCL2 gradient, leading to their infiltration into the aortic wall, where they contribute to the chronic inflammatory environment that drives aneurysm formation and progression (Figure 1) (17, 18). This chemokine-mediated recruitment of inflammatory cells can set the stage for the cellular and molecular remodeling events central to AAA pathology.

The CCR2 receptor binding to its principal ligand, CCL2, triggers a cascade of intracellular signaling pathways, including ERK1/2, PI3K/Akt, JAK/STAT, and NF-κB (Figure 1) (19, 20). These pathways converge on key pro-inflammatory and tissue remodeling responses. Upon activation, CCR2 also promotes monocyte migration and activation, leading to the release of MMP-2 and MMP-9, reactive oxygen species, and pro-inflammatory cytokines (Figure 1) (21–23). These MMPs present in aneurysmal tissue degrade the ECM, with collagen type I/IV, laminin, and E-cadherin serving as substrates (24). Moreover, CCR2 signaling is thought to affect fibroblast and VSMC behavior, contributing to phenotypic switching, apoptosis, and further weakening of the aortic wall (Figure 1) (22). These mechanisms are critical in AAA pathogenesis, as persistent CCR2 activity sustains a chronic inflammatory state that drives ECM breakdown, wall thinning, and expansion of the aneurysmal segment. Subsequent inflammation and cell activation lead to further secretion of MCP-1, promoting additional monocyte recruitment, occasionally becoming a circled loop of inflammation that cannot be well-balanced by anti-inflammatory mediators (22, 24, 25). This excessive inflammatory activation is believed to promote AAA progression and contributes to its eventual rupture, despite the tissue’s attempt to repair the wall.

### CCL2/CCR2-targeting strategies in AAAs

Several animal studies have underscored the pivotal role of the CCL2/CCR2 axis in the initiation and progression of aortic aneurysms (25–30). Transplantation of *Ccr2^−/−^* bone marrow into *Apoe^−/−^* mice was sufficient to mitigate aneurysm formation following aneurysm induction with an angiotensin II (ANG II) infusion model. Despite the upregulation of CCL2 in the aortic tissue, macrophage infiltration and the levels of inflammatory cytokines IL-1β and IL-6 remained persistently low. These results implicate bone marrow-derived CCR2+ macrophages as key drivers of inflammation and cytokine release, thereby contributing to aneurysm development and progression (25).

Building on these findings, subsequent studies investigated the impact of whole-body *Ccr2* knockout on AAA formation. MacTaggart et al. examined the roles of CCR2, CXCR3, and CCR5 in a murine CaCl2-induced infrarenal AAA model (26). This study demonstrated that only the *Ccr2^−/−^* cohort had significant inhibition of AAA formation, resulting in smaller aneurysm diameters compared to the CXCR3 and CCR5 knockout groups. These findings highlight that CCR2 is unlikely to be a non-specific bystander and rather drives molecular mechanisms that progress AAA disease pathology (26). Additionally, a histological analysis of *Ccr2^−/−^* aortic tissue revealed a significant reduction in macrophage infiltration and decreased levels of MMP-2 and MMP-9, again underscoring the critical role of CCR2 in driving AAA pathology (26). Similarly, Daugherty et al. demonstrated that a whole-body deficiency of *Ccr2* also attenuated ascending and suprarenal aortic formation in the *Apoe^−/−^* and ANG II model (27).

Recent studies have focused on the critical role of CCR2 in AAA rupture. Whole-body genetic knockdown of *Ccr2* in a mouse model prone to AAA rupture, achieved using intraluminal porcine pancreatic elastase (PPE), daily β-aminopropionitrile (BAPN), and ANG II, demonstrated a significantly reduced incidence of infrarenal AAA rupture, confirming the pivotal role of CCR2 in aneurysm expansion leading to rupture (28). Given this critical role, recent investigations have also used 64Cu-DOTA-ECL1i, a radiolabeled CCR2-targeting peptide. Using positron emission tomography (PET), 64Cu-DOTA-ECL1i can be used to assess CCR2 content in rupture-prone AAAs following induction with intraluminal PPE and BAPN in male and female Sprague Dawley rats (29, 30). These studies showed the specificity of CCR2 tracing in both rat AAAs and human explanted AAA tissue, highlighting its potential utility as a theranostic tool in humans *in vivo*.

Further investigations evaluated the efficacy of RS504393, a specific CCR2 pharmacological inhibitor (30). When introduced on day 3, after the establishment of AAA through intraluminal elastase exposure, RS504393 treatment significantly reduced both AAA size and rupture events in male and female rats. Inhibition of CCR2 also led to notable decreases in inflammatory cytokines, chemokines, elastin fragmentation, and macrophage infiltration. A dose-dependent response was observed, with continued treatment further reducing AAA progression, rupture, and immune cell infiltration (30). An additional study explored the impact of a ketogenic diet on AAA in rats. This nutritional intervention significantly mitigated AAA rupture and significantly downregulated CCR2 content, MMP activation, and preserved elastin with an overall improved balance in the extracellular matrix (28). These studies provided significant insights into the molecular mechanisms driving AAA pathology and highlighted the potential of targeting the CCL2/CCR2 axis as an effective therapeutic strategy for AAA stabilization.

### Targeting CCR2/CCL2 in other pathologies

In addition to the demonstrated utility of CCR2 targeting in AAA models, CCR2/CCL2 axis modulation has also been investigated as an inhibitor of fibrosis, serving as a potential target for increasing the efficacy of chemotherapy delivery (31–39). This has led to the development and use of several therapeutic agents in pre-clinical animal models and in larger-scale clinical trials more recently (40–46).

Since fibrosis in the aneurysmal aortic wall in animal models and human tissue has likewise been identified as a pathological factor for potential intervention (31, 32), targeted CCR2 inhibition to mitigate macrophage-mediated end-organ fibrosis has also been evaluated in murine models. In an acute myocardial infarction model, CCR2 inhibition using inhibitor 4 (Teijin compound 1), which was applied directly to a caged nitric oxide donor patch, shifted macrophages to an anti-inflammatory M2 polarization and improved post-infarction cardiac function (33). This inhibitor was additionally explored through liposomal coupling for VCAM1-directed endothelial targeting for drug delivery as a means to mitigate monocyte transmigration, with demonstrated efficacy in reducing macrophage recruitment to *Apoe**^−/−^* murine aortic tissue (34). Cenicriviroc (CVC) is an oral dual antagonist of CCR2/CCR5 originally designed for the treatment of Human Immunodeficiency Virus and has been found to attenuate hepatic fibrosis in a murine injury model (35, 36). Through RNA sequencing and bulk-RNA analysis, it was observed that CVC led to the downregulation of common profibrotic signaling pathways (JAK-STAT, TNF-NFκB, and MAPK-ERK), with collagen I immunofluorescence demonstrating a similar level of expression to control livers.

CCR2 inhibition has been further explored as an adjunct to chemotherapy targeting PD-1. Three oral inhibitors—CCX872, MK0812, and RS504393—have been explored in murine glioblastoma and breast cancer metastasis models (37–39). These studies demonstrated a reduction in tumor myeloid-derived suppressor burden, with evidence that these cells were retained within the bone marrow. Therapeutically, CCR2 inhibition was found to provide a survival benefit when co-administered with anti-PD-1 chemotherapy, whereas anti-PD-1 therapy alone was insufficient. These studies did not report any notable toxic effects of drug administration.

### Targeted CCR2/CCL2 axis inhibition in human trials

Clinical translation of CCR2 inhibitors has been limited to date. However, in 2011, a study investigated the use of MLN1202 (plozalizumab), a monoclonal antibody that interacts with CCR2 to inhibit CCL2 binding. Including 112 patients randomized to MLN1202 or placebo, the study achieved its primary endpoint of reducing serum C-reactive protein in patients at high atherosclerotic risk (40). CCR2 inhibition with cenicriviroc for the mitigation of hepatic fibrosis was explored in the AURORA phase III study, a randomized trial involving 1,778 patients (41). While this study demonstrated a good safety profile with only two patients experiencing treatment-related adverse events warranting medication discontinuation, cenicriviroc did not demonstrate efficacy in preventing non-alcoholic steatosis (NASH) (41). PF-04136309 is an orally administered CCR2 inhibitor that was recently investigated in a phase 1b study in 21 patients as an adjuvant to the standard chemotherapeutic agents used in patients with pancreatic ductal adenocarcinoma (42). This study noted potential pulmonary toxicity related to CCR2 inhibitor administration, though this was confounded by the concurrent use of adjunct chemotherapeutic agents. A summary of completed human clinical trials utilizing CCR2 antagonist therapy is presented in Table 1 (40–46).

**Table 1:** CCR2 antagonists used in completed human trials, with the drug and dosing characteristics and the pathology of interest investigated in each trial.

Compound	Characteristics	Disease studied	Dosing	Trial phase/type	References
CCX140-B	Small molecule, selective CCR2 antagonist	Diabetic nephropathy	5 mg or 10 mg PO daily	Phase 2, RCT, double-blind	(43)
BMS-813160	Small molecule, dual CCR2/CCR5 antagonist	Pancreatic ductal adenocarcinoma	Up to 300 mg PO BID	Phase 1/2	(44)
MLN1202 (plozalizumab)	Humanized monoclonal antibody, anti-CCR2	Melanoma, cardiovascular disease	2–10 mg/kg	Phase 1/2	(40, 45)
Cenicriviroc (CVC)	Small molecule, dual CCR2/CCR5 antagonist	NASH, liver fibrosis	150 mg PO daily	Phase 3/RCT	(41)
JNJ-41443532	Small molecule, selective CCR2 antagonist	Type 2 diabetes mellitus	100 mg PO daily	Phase 2/RCT	(46)
PF-04136309	Small molecule, selective CCR2 antagonist	Pancreatic ductal adenocarcinoma	Up to 750 mg PO BID	Phase 1b	(42)

## Discussion

Herein, we provided an updated review of recent pre-clinical assessments and early human clinical translation of therapeutics targeting the CCR2/CCL2 axis—particularly in the setting of AAA management. Recent evidence in AAA biology suggests that CCR2 may be a cellular marker for disease progression. Investigators have found that PET/CT uptake for CCR2 content is not only higher in AAA patients vs. controls, meaning it is disease-specific, but it also correlates with areas that are prone to rupture based on a finite element analysis of mechanical wall stress. These findings, along with the specificity of CCR2 in AAA human tissues and MCP-1 expression, suggest a potential theranostic role for CCR2 in AAA. Animal studies of CCR2 knockout or inhibition have demonstrated utility in mitigating AAA rupture. Ongoing assessments of CCR2 inhibitor compounds have suggested reasonable *in vivo* biotolerance without substantial adverse effects in both animal and early human clinical trials investigating other pathologies. Similar to oncological studies, vascular therapies are moving towards more specific, cellular-driven treatments to improve patients' outcomes while minimizing off-target effects.
